# Validating and updating a risk model for pneumonia – a case study

**DOI:** 10.1186/1471-2288-12-99

**Published:** 2012-07-20

**Authors:** Ulrike Held, Daniel Sabanes Bové, Johann Steurer, Leonhard Held

**Affiliations:** 1 Horten Centre for Patient Oriented Research and Knowledge Transfer, University Hospital Zurich, Zurich, Switzerland; 2 Institute for Social and Preventive Medicine, Division of Biostatistics, University of Zurich, Zurich, Switzerland

**Keywords:** Validation, Predictive performance, Bayesian model, g-factor, Pneumonia

## Abstract

**Background:**

The development of risk prediction models is of increasing importance in medical research - their use in practice, however, is rare. Among other reasons this might be due to the fact that thorough validation is often lacking. This study focuses on two Bayesian approaches of how to validate a prediction rule for the diagnosis of pneumonia, and compares them with established validation methods.

**Methods:**

Expert knowledge was used to derive a risk prediction model for pneumonia. Data on more than 600 patients presenting with cough and fever at a general practitioner’s practice in Switzerland were collected in order to validate the expert model and to examine the predictive performance of it. Additionally, four modifications of the original model including shrinkage of the regression coefficients, and two Bayesian approaches with the expert model used as prior mean and different weights for the prior covariance matrix were fitted. We quantify the predictive performance of the different methods with respect to calibration and discrimination, using cross-validation.

**Results:**

The predictive performance of the unshrinked regression coefficients was poor when applied to the Swiss cohort. Shrinkage improved the results, but a Bayesian model formulation with unspecified weight of the informative prior lead to large AUC and small Brier score, naïve and after cross-validation. The advantage of this approach is the flexibility in case of a prior-data conflict.

**Conclusions:**

Published risk prediction rules in clinical research need to be validated externally before they can be used in new settings. We propose to use a Bayesian model formulation with the original risk prediction rule as prior. The posterior means of the coefficients, given the validation data showed best predictive performance with respect to cross-validated calibration and discriminative ability.

## Background

Over the past decade, there has been increasing interest in the development and validation of prediction models for disease risk. Knowledge about multiple predictor variables and their weights enables physicians to estimate the risk of disease presence [1-4]. This study focuses on the clinical diagnosis of pneumonia, which is a concern when a patient presents with recent onset of cough and fever to a general practitioner (GP). Based on these symptoms and the results of physical examination, the doctor needs to decide on further testing, e.g., chest radiography and/or antibiotic treatment.

In 2008, Miettinen et al. [5] derived a probability function for pneumonia by developing 36 hypothetical cases (called vignettes) based on a set of 25 clinical-diagnostic indicators. In contrast to the common approach of developing a risk prediction model with a set of patients in an epidemiologic study, the judgement of a set of medical experts was the basis for this diagnostic probability function. Twenty-two clinical experts independently assigned risk probabilities to these 36 vignettes and a logistic function of 14 of these diagnostic indicators was fitted to the median probabilities (expert model). A more detailed description of the development of the expert model can be found in the Appendix.

The aim of this study is to validate and update this expert model. Data on more than 600 patients presenting with cough and increased body temperature at a GP practice in Switzerland were collected between 2006 and 2009 [6]. Information about all 14 clinical-diagnostic indicators identified by the expert panel was collected. In addition to that, the GP’s diagnosis of pneumonia is confirmed through chest radiography by experienced radiologists, which serves as a reference standard.

We compare five different approaches to combine the results of the expert model with those of the Swiss cohort study. The predictive performance of the different approaches is measured with respect to discrimination, calibration and overall accuracy of prediction as measured by the Brier score. We assessed these quantities in a naïve way, and by leave-one-out cross validation.

## Methods

When a patient presented with fever and cough at a participating GP, he was asked to take part in this validation study (Swiss cohort). After obtaining written informed consent for participation data on the set of diagnostic indicators was assessed. These indicators included the following 14 variables: age, duration of new or worsened cough (days), maximum temperature, dyspnea, dyspnea at effort only, rigors, smoking (number of cigarettes per day), current temperature, signs of upper respiratory infection, prolonged expiration, percussion dullness, auscultation friction rub, auscultation diminished inspiration sound and auscultation abnormality breath sound. The expert model includes quadratic terms for four variables, while the other variables enter in a linear fashion [5]. The dependent variable in the Swiss cohort was pneumonia (yes/no) and the GP’s diagnosis was confirmed through chest x-ray.

### Imputation of auscultation friction rub

A large number (46%) of observations were missing for the variable “auscultation friction rub” (AFR) in this cohort because the first version of the questionnaire accidently did not ask for this variable. We imputed the missing values of this indicator in the following way: we fitted a logistic regression model to the available observations of the binary variable AFR, including all predictors of the expert model. Based on this model, we obtained the predicted probabilities for the missing observations of AFR. In order to dichotomise these probabilities, we chose the cut-off at 0.16 to keep the overall prevalence of AFR at 5%.

### Performance measures

We measure the predictive performance of the probability functions for pneumonia by their discriminative ability, calibration and overall accuracy of prediction. The discriminative ability of a risk model is typically measured by the area under the receiver operating characteristic curve (AUC). For prediction models that can distinguish well between high and low risk patients, the AUC will be close to 1. For calibration, we visually assess how well the model-based probabilities for pneumonia agree with the observed outcomes.

When prediction models are applied to a new data set, the predicted probabilities are typically too extreme [7] and calibration plots with pointwise 95% confidence intervals can depict this problem. The calibration slope shows the amount of shrinkage needed for a model.

The Brier score (BS) is an overall measure of accuracy, as it is a combination measure of discrimination and calibration. The Brier score, or average prediction error is defined as follows:

(1) $$\text{BS}=\frac{1}{n}\sum_{i=1}^{n}{\left(p_{i}-y_{i}\right)}^{2},\text{i}=1,\ldots ,\text{n}\text{,}$$

with p_i_ : predicted probabilities by the model, y_i_ : observations (0 or 1), and n: number of observations. Models with smaller Brier score are to be preferred.

The Brier score has two different reference values. One depends on the prevalence $\pi =\frac{1}{n}\sum_{i=1}^{n}y_{i}$ of the event (here pneumonia) in the study population. It is calculated by setting p_i_ = π for all i, so BS = π(1-π). The other one corresponds to coin flipping, and it is calculated by setting p_i_ = 0.5 for all i, so BS = 0.25. A useful prediction rule ought to have a Brier score smaller than 0.25 and ideally also smaller than π(1-π) ≤ 0.25. Bradley et al. [8] propose a method to estimate confidence intervals for Brier scores, which was used here.

### Description of the approaches

#### Approach 1

We use the coefficients β_exp_ given in the expert model by Miettinen et al., and apply them directly to the Swiss cohort to obtain the predicted pneumonia risk probabilities p = X β_exp_, where X is the matrix of regressor variables of the Swiss cohort.

#### Approach 2

The coefficients of the expert model are likely subject to overfitting, as there were 25 diagnostic indicators originally under examination, but only 36 vignettes. To quantify the amount of overfitting, we determine the shrinkage factor by studying the calibration slope b when fitting the logistic regression model [9,10], page 272]:

(2) $$\text{logit}\left(\text{P}\left(\text{Y}=1\right)\right)=\text{a}+\text{b}*\text{logit}\left(\text{p}\right)\text{,}$$

where p is the vector of predicted probabilities. The slope b of the linear predictor defines the shrinkage factor. Well calibrated models have b ≈ 1. Thus, we recalibrate the coefficients of the genuine expert model by multiplying them with the shrinkage factor (shrinkage after estimation).

#### Approach 3

An alternative to incorporate shrinkage is to use a Bayesian approach and to shrink the regression coefficients during estimation. We specify a Bayesian logistic regression model with an informative Gaussian prior distribution β ~ N(β_exp_ , Σ_exp_) for the regression parameter vector β, assuming that the prior mean β_exp_ are the coefficients from the expert model. We derive Σ_exp_ directly from Miettinen et al. paper in the following way:

(3) $$\sum_{\exp}={\overset{⌢}{\sigma }}^{2}{\left(X^{T}X\right)}^{-1},\;\text{where}\;{\overset{⌢}{\sigma }}^{2}=\frac{1}{m-p-1}\sum_{i=1}^{m}{\left(y_{i}-{\overset{⌢}{y}}_{i}\right)}^{2}\text{.}$$

There were m = 36 vignettes in the expert study.

We solve the model using integrated nested Laplace approximations (INLA), a recently developed approach to statistical inference for latent Gaussian Markov random field models [11]. An advantage of the INLA approach to Bayesian inference is its speed and its accurate approximations to the marginal posterior distributions of the hyper-parameters and latent variables. It also provides cross-validated predicted probabilities without the need to refit the data [12].

#### Approach 4

We can increase the flexibility of the Bayesian Approach 3 by introducing an additional unknown factor g in the prior covariance matrix: β ~ N(β_exp_ , g*Σ_exp_). We assume that the parameter g is a priori unknown, following a chi-squared distribution with one degree of freedom, thus having mean 1 and variance 2. Other choices are possible, of course. The parameter g is a measure for the prior-data conflict: the larger g is a posteriori, the larger is the discrepancy between the prior and the data, and the weight of the prior distribution on the posterior becomes small. The reference value g = 1 corresponds to Approach 3 and no prior-data conflict. This extended Bayesian approach can also be fitted with INLA.

#### Approach 5

In this approach we ignore the prior knowledge derived from the expert model, and estimate the regression coefficients directly from the Swiss cohort. This means, we use the Swiss cohort for derivation and validation of the model. We introduce Approach 5 to show the complete spectrum from “only prior knowledge (Approach 1)” to “no prior knowledge (Approach 5)”.

### Cross validation

We compare the five approaches with respect to the estimated regression coefficients, AUC, calibration plots and Brier score in a naïve way. Additionally, we compute AUC and Brier score with leave-one-out cross validation for the Swiss cohort. Specifically, this means for Approach 2 that we repeat the estimation of the shrinkage factor n times, each time leaving out the i-th observation, subsequently applying the shrinkage factor estimated from the remaining n-1 observations to β_exp_ for the predicted probability for the omitted observation i. In this way we obtain a cross-validated vector of fitted values for each observation, and this is used for the calculation of AUC and the Brier score. In Approaches 3 and 4, INLA software directly allows calculation of cross-validated AUC and the Brier score, see Held et al. [12] for details. In Approach 5 we derived the cross-validated results by fitting the model n times to n-1 observations, each time estimating the regression coefficients based on n-1 observations and predicting the i-th omitted observation.

All analyses were performed with R 2.14.2 [13] and INLA [11].

## Results

In the Swiss cohort study, data on 621 patients presenting with cough and elevated body temperature at their GP were collected. Of these 621 patients, 127 had radiographic signs of pneumonia (π = 20.5%). After imputation of the variable AFR, 490 patients had complete (or imputed) observations in all of the 14 predictor variables of the expert model. Details about the Swiss cohort data can be found elsewhere [6].

**Figure 1:**
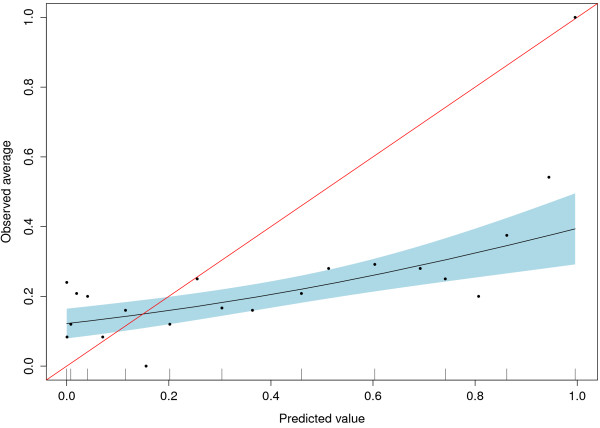
**Calibration plot with 95% confidence interval for Approach 1**.

In Approach 1, the published coefficients of the genuine expert model were applied to the Swiss cohort data. The resulting AUC was 0.633 (95% CI: 0.567 − 0.695), showing that the discriminative ability of this approach is rather low. To answer the question whether the predicted probabilities correspond to the observed probabilities, Figure 1 shows the calibration of the results. There turns out to be mis-calibration between the predicted and the observed risks, with underprediction in the range < 0.2, and overprediction > 0.2 as one typically sees for overfitted original models . The Brier score of Approach 1 is 0.246 (0.219-0.272). It is larger than the prevalence based reference value of 0.163, but smaller than 0.250 from coin-flip. The cross-validated results are identical here since the expert model was simply applied to the Swiss cohort.

**Figure 2:**
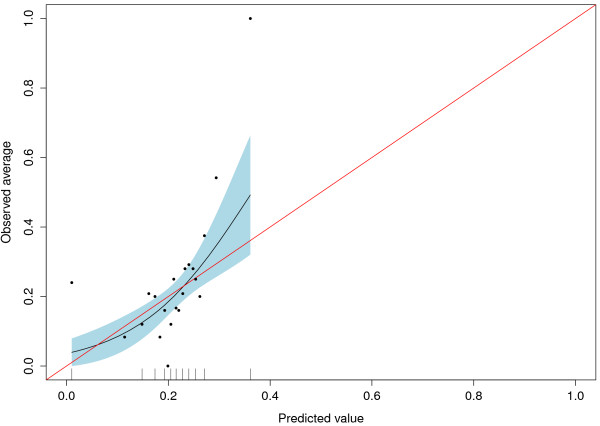
**Shrinked regression coefficients with 95% confidence interval for Approach 2**.

**Figure 3:**
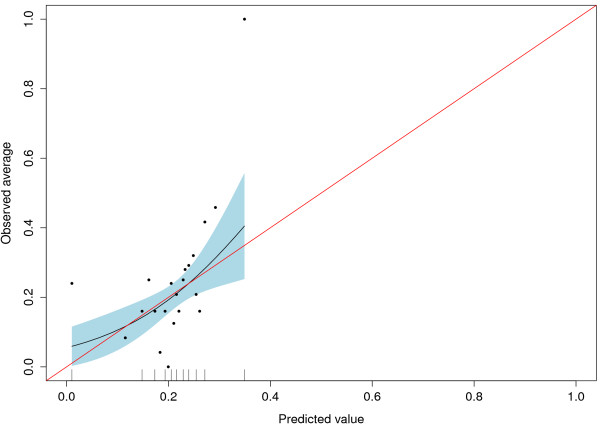
**Cross-validated shrinked coefficients with 95% confidence interval for Approach 2**.

The mis-calibration of Approach 1 indicated the need for re-calibration and we obtained a uniform shrinkage factor when we fitted logit(P(Y = 1)) = a + b*logit(p) in Approach 2. We obtained the estimates a = −1.20 and b = 0.11, indicating heavy shrinkage. The corresponding AUC is not affected by re-calibration, but Figure 2 shows the improved agreement between predicted and observed risks of the re-calibrated expert model. The naïve Brier score for Approach 2 is 0.163. When we cross-validated the shrinkage factor of the regression coefficients, the resulting AUC was 0.616 (0.550-0.679) and the corresponding Brier score was 0.164 (0.143-0.185). Figure 3 shows the calibration plot for the cross-validated and re-calibrated regression coefficients.

For Approach 3, the results of the expert model were used as prior distribution for the coefficients in a Bayesian framework. Using the estimated covariance matrix from the Miettinen et al. paper, the naïve AUC was 0.759, considerably larger than that of the previous two approaches. The Brier score of Approach 3 was 0.135, and thus smaller than both reference values. The cross validated AUC was 0.707 (0.644-0.763) and corresponding Brier score was 0.146 (0.126-0.167).

In Approach 4, where g scales the covariance matrix to allow for more flexibility, the AUC was 0.757, and the Brier score was 0.136, these values are comparable to those of Approach 3. The posterior mean of g was 0.79 (0.45-1.73), indicating no substantial prior-data conflict for this application. When we cross-validated the results, the AUC dropped to 0.707 (0.644-0.763) and the Brier score to 0.146 (0.126-0.166).

Finally, we applied Approach 5, ignoring the expert prediction model. Unsurprisingly, this lead to the largest naïve AUC of 0.765 and smallest Brier score of 0.134. However, the cross-validated results were worse, with AUC of only 0.695 (0.632-0.752) and BS of 0.149 (0.128-0.171).

Naïve comparison of Approaches 1 through 5, ignoring the problem of over-fitting, shows that Approach 5 is the one with the largest AUC and smallest Brier score. With respect to the cross-validated results, we can see from a decrease in AUC and corresponding increase in Brier score that there is optimism for all approaches. However, Approaches 3 and 4 are the most preferable ones. Although Approaches 3 and 4 give very similar results for the combination of expert knowledge and new data, we prefer Approach 4 as it gives more flexibility in the case that there is more prior-data conflict. The predictive performance (AUC and Brier score, with 95% confidence intervals) of all five approaches, naïve and cross validated, is given in Table 1.

**Table 1:** Naïve and cross-validated comparison of the predictive performance of the five approaches, with 95% confidence intervals

**Comparison**	**Statistic**	**Approach 1**	**Approach 2**	**Approach 3**	**Approach 4**	**Approach 5**
Naïve	AUC*	0.633 (0.567-0.695)	0.633 (0.567-0.695)	0.759 (0.701-0.809)	0.757 (0.699-0.807)	0.765 (0.707-0.815)
	BS**	0.246 (0.219-0.272)	0.163 (0.142-0.183)	0.135 (0.116-0.154)	0.136 (0.116-0.155)	0.134 (0.114-0.154)
Cross validated	AUC	0.633 (0.567-0.695)	0.616 (0.550-0.679)	0.707 (0.644-0.763)	0.707 (0.644-0.763)	0.695 (0.632-0.752)
	BS	0.246 (0.219-0.272)	0.164 (0.143-0.185)	0.146 (0.126-0.167)	0.146 (0.126-0.166)	0.149 (0.128-0.171)

* AUC = Area under the receiver operating characteristic curve, ** BS = Brier score.

Table 2 shows the regression coefficients of the most preferable Approach 4 compared to the original coefficients from the expert model (Approach 1) and the maximum likelihood results (Approach 5). The regression coefficients of Approaches 4 and 5 are relatively similar in contrast to those of Approach 1.

**Table 2:** Estimated regression coefficients: original expert model (Approach 1) compared to Approaches 4 and 5

**Variable**	**Approach 1**	**Approach 4**	**Approach 5**
	**Expert model**	**Random g**	**Maximum likelihood**
Intercept	−79.19	−37.50	−36.31
Age	−0.0054	−0.0017	−0.0079
Duration of new/worsened cough	0.15	0.027	0.033
Maximum temperature	1.89	0.39	0.37
Dyspnea	−1.34	0.51	0.59
Dyspnea at effort only	2.63	−0.078	−0.11
Rigors	0.52	−0.14	−0.017
Number of cigarettes / day	0.023	0.017	0.0054
Current temperature	0.14	0.54	0.53
Signs of upper respiratory infection	1.23	−0.43	−0.61
Prolonged expiration	−1.40	0.12	0.16
Percussion dullness	0.84	1.44	1.60
Auscultation friction rub	0.97	1.39	1.41
Auscultation diminished insp. sound	0.41	0.69	0.44
Auscultation abnormality breath sound	0.52	0.66	0.73
(Age – 45)^2^	0.00017	0.00050	0.00058
(Duration of cough – 10)^2^	−0.00912	−0.0010	−0.00083
(Maximum temperature – 38.5)^2^	−0.83	0.057	0.046
(Current temperature – 38.5)^2^	−0.33	0.033	0.072

## Discussion

In this study, we validated a published risk prediction model for the presence of pneumonia. The genuine prediction model was based on expert opinion rather than on an epidemiologic study with real patient data. For this reason, we consider the expert model to be of very high quality. In this validation study, we used data of 490 complete (and imputed) cases of patients presenting with cough and elevated body temperature at their GP in Switzerland, with information on all variables of the expert model. We introduced five approaches, ranging over the whole spectrum whether or not to include the expert knowledge. We started with an approach based on prior knowledge from the expert model, concluding with an approach ignoring prior knowledge. Two of the approaches were Bayesian, one of them giving us the possibility to quantify the presence of a prior-data conflict. In a naïve comparison, ignoring the problem of over-fitting, we found that the approach ignoring the expert knowledge performed best on the Swiss cohort (Approach 5), i.e. it was the one with largest AUC and smallest Brier score. When the performance of the approaches was investigated in an out-of-sample prediction using leave-one-out cross-validation, however, the most favourable approaches were the two Bayesian approaches. The Bayesian model formulation with informative prior based on the expert knowledge and with a flexible prior weight of the expert knowledge lead to the most favourable results. In this analysis we found no prior-data conflict because the posterior mean of the g-factor was 0.79 and the 95% confidence interval includes 1. This means the prior weight is more or less unchanged, compared to the data having weight 1.

As the advantage of the flexibility in Approach 4 compared to Approach 3 is not clearly visible in this application, we conducted some additional simulation study by increasing the discrepancy between prior and data. In one scenario we divided the covariance matrix by five and in a second scenario we multiplied the prior vector with factors uniformly distributed on the interval [0.5;2]. We found that the effect of these changes on g was large: in the first scenario the posterior mean of g was 3.5, and in the second scenario it was even 14.5. So for applications in which the quality of the prior is questionable, the introduction of additional flexibility appears to be necessary.

### Our findings in the context of existing evidence

The development of multi-variable clinical prediction rules has become rather popular in recent years [1], but unless there is evidence that they work well on other patients than in those of the initial development set they should not be used in clinical practice [14]. Our study confirms that the amount of optimism is large when regression models are fit to a data set without validation. Especially when the sample size is small or the number of covariates is large, as is the case in the derivation set of our presented expert model, shrinkage is a serious problem [15,16]. Due to such a large variety of potential reasons for poor performance of prediction models, the reservations of clinicians to use them in routine clinical practice [17-19] is understandable. With the two Bayesian models, we proposed two alternative methods for the validation of over-fitted models that add transparency to the whole process. New or old data as prior can be used to improve the performance of the prediction model, but - when using Approach 4 - with the additional flexibility to down-weight this information if the observed data and the prior differ too much. Moreover by studying the g-factor we can learn about the influence of the prior, and whether it is useful in the specific application.

### Strengths and limitations

The strength of our study is the availability and integration of data on all variables of the published prediction model in the majority of the patients. Moreover, the collection of data in the Swiss cohort was guided by the intent to validate the risk model derived from the experts.

A limitation of this study is the discrepancy between pneumonia prevalence in the derivation and the validation data. The vignettes of the expert model were constructed with emphasis on low-probability cases, but the prevalence of pneumonia in the Swiss cohort was relatively high (20.5%), especially higher than one would expect in a GP setting where the prevalence ranges between 3% and 20% [20]. This discrepancy makes it more difficult for the risk prediction rule to perform well, but we note that this inhomogeneity between derivation and validation population is a common problem in validation studies [10] [page 345].

We have not pursued further developments of Approach 5, as could be bootstrapping or related methods, in order to improve the performance of the model developed in the Swiss cohort in the cross-validation setting.

### Implications for practice

We showed that the expert prediction model for pneumonia was mis-calibrated when applied to the Swiss cohort. The under-estimation of the true probability of pneumonia could – in the worst case – lead to a missed pneumonia case, with considerable potential for harm of the patient. In the other direction when a patient is falsely diagnosed with pneumonia he will be prescribed antibiotics, which has a negative implication not only for him but also for the health care system due to potential bacterial resistance and higher cost. We propose to use the adjusted regression coefficients for the prediction of pneumonia resulting from Approach 4, especially in settings with similar prevalence of pneumonia as in this one. The reduction of necessary chest x-rays could be a useful implication for practice.

### Implications for research

Further studies should be undertaken in the evaluation of the prior-data conflict. It would be helpful for future research, to analyse factors influencing the magnitude of the prior-data conflict g. In the Bayesian framework, there is ongoing discussion about the choice of the prior distribution. Some statisticians believe that the use of non-informative priors is preferable, and to let the data speak for themselves. On the other hand, if prior information is available, it should clearly be used. By introducing the g-factor, the Bayesian model has the flexibility to down-weight the prior knowledge, indicating that there is a discrepancy between the derivation and the validation model.

## Conclusions

Published risk prediction rules in clinical research need to be validated before they can be used in new settings. We propose to use a Bayesian model formulation with the original risk prediction rule as prior. The posterior means of the coefficients, given the validation data, and allowing for flexible weight of the original prediction rule, turn out to have largest discriminative ability, and lowest mis-calibration, measured by the AUC and the Brier score. When compared to the un-validated coefficients from the expert model, we found considerable differences.

## Appendix

## Description of the probability function of pneumonia

Miettinen et al. [5] developed a probability function to address the concern of a clinical diagnosis of pneumonia when a patient presents with recent cough and fever at a general practitioner’s practice. This probability function is based on expert knowledge rather than on an epidemiologic study. For this, a set of 36 hypothetical case presentations was specified and each member of a medical expert panel set the corresponding probability for pneumonia in each of these cases. The case presentations were based on a set of 25 diagnostic indicator variates. For each of the individual vignettes Miettinen et al. calculated a median probability over all 22 experts, and fitted a linear model of the diagnostic indicators to the logit of the median probabilities. Variates were excluded from the final probability function one at a time if they did not change the score value by more than ± 0.2 at most. The resulting final probability function is based on a set of 14 different variables, with four of them entering in a quadratic form. The estimated coefficients of this final probability function are given in Table 4^2^.

## Competing interests

Financial competing interests

- In the past five years have you received reimbursements, fees, funding, or salary from an organization that may in any way gain or lose financially from the publication of this manuscript, either now or in the future? Is such an organization financing this manuscript (including the article-processing charge)? If so, please specify.

UH, DSB, JS, LH: none declared.

- Do you hold any stocks or shares in an organization that may in any way gain or lose financially from the publication of this manuscript, either now or in the future? If so, please specify.

UH, DSB, JS, LH: none declared.

- Do you hold or are you currently applying for any patents relating to the content of the manuscript? Have you received reimbursements, fees, funding, or salary from an organization that holds or has applied for patents relating to the content of the manuscript? If so, please specify.

UH, DSB, JS, LH: none declared.

- Do you have any other financial competing interests? If so, please specify.

UH, DSB, JS, LH: none declared.

Non-financial competing interests

- Are there any non-financial competing interests (political, personal, religious, ideological, academic, intellectual, commercial or any other) to declare in relation to this manuscript? If so, please specify.

UH, DSB, JS, LH: none declared.

## Author’s contributions

UH was responsible for the conception and design of this study, carried out parts of the statistical analysis and drafted and revised the manuscript. DSB carried out the statistical analyses involving INLA and revised the manuscript. JS was responsible for the acquisition of data, participated in the concept of the study and interpretation of the results. LH made substantial contributions to the design of this study and data analysis including interpretation of the results and revised the manuscript. All authors read and approved the final manuscript.

## Pre-publication history

The pre-publication history for this paper can be accessed here:

http://www.biomedcentral.com/1471-2288/12/99/prepub
